# Maternal Supplementation with Dietary Betaine during Late Gestation Increased Ewe Plasma Creatine and Lamb Thermoregulation under Field Conditions

**DOI:** 10.3390/ani14172605

**Published:** 2024-09-07

**Authors:** Billie-Jaye Brougham, Alice C. Weaver, Alyce M. Swinbourne, Megan R. Tscharke, Amy L. Munn, Jennifer M. Kelly, David O. Kleemann, William H. E. J. van Wettere

**Affiliations:** 1 Davies Livestock Research Centre, School of Animal and Veterinary Sciences, The University of Adelaide, Roseworthy, SA 5371, Australia; billie-jaye.brougham@adelaide.edu.au (B.-J.B.); megan.tscharke@adelaide.edu.au (M.R.T.); amy.munn@adelaide.edu.au (A.L.M.); 2 South Australian Research and Development Institute, Turretfield Research Centre, Rosedale, SA 5350, Australia; alice.weaver@sa.gov.au (A.C.W.); alyce.swinbourne@sa.gov.au (A.M.S.); jenkelly@internode.on.net (J.M.K.); dave.kleemann@sa.gov.au (D.O.K.)

**Keywords:** betaine, supplementation, gestation, plasma creatine, lamb, survival, thermoregulation

## Abstract

**Simple Summary:**

Perinatal twin lamb mortality reduces productivity and reproductive efficiency in Australian sheep meat and wool enterprises. This study investigated whether supplementing 4 g of betaine into the diets of twin-bearing Merino ewes during late pregnancy would improve lamb viability measures and survival to weaning under field conditions. The results indicated that dietary supplementation with betaine increased plasma creatine concentrations in ewes at day 130 of gestation and lamb rectal temperature within 4–24 h following birth. While the findings of this study provide some new, promising results, more research in the field is required before supplementation with betaine during late gestation can be used as an on-farm strategy to improve lamb viability and survival.

**Abstract:**

Twin lamb mortality is a significant economic problem impacting the Australian sheep industry. Maternal betaine supplementation improved lamb vigour and early post-natal survival when ewes and lambs were housed indoors, suggesting that betaine may be beneficial to feed under extensive pasture systems. This study investigated whether maternal betaine supplementation during late gestation would improve Merino twin lamb live weight, thermoregulation, vigour and survival to weaning under field conditions. Ewes received dietary betaine at either 0 g/day (CTL; *n* = 115) or 4 g/day from day 110 of gestation (dG 110) until ~49 days post-partum (pp) (BET; *n* = 115). Measures indicative of lamb viability and survival were collected within 4–24 h of birth and at ~49 days pp and ~93 days pp. BET ewes had higher creatine and creatinine concentrations at dG 130 than CTL ewes (*p* < 0.05). BET lambs had a higher rectal temperature within 4–24 h following birth than CTL lambs (*p* < 0.05). CTL lambs were heavier at ~49 days pp and grew faster from birth to ~49 days pp than BET lambs (both *p* < 0.05). The time taken after release from the researcher to first suckling was quicker in the CTL lambs than BET lambs (*p* < 0.05). This study demonstrated that supplementing betaine increased creatine concentration in twin-bearing ewes and thermoregulatory capacity in neonatal lambs under extensive grazing systems.

## 1. Introduction

The Australian sheep flock consists of 40.7 million breeding ewes, with 30.4 million (75%) of these being Merinos due to high-quality wool production [1,2]. Most Merino enterprises lamb on pasture during winter/spring and receive little to no supervision during lambing in order to maximise the ewe-lamb bond [3]. One major problem the industry is currently facing are high lamb mortality rates, where approximately 12 million twin lambs die prior to weaning per year, limiting output and representing a major welfare concern [4,5,6,7]. Lamb deaths are most common during the first three days of life [4,6,7,8], with Merino twin-born lambs being 2–2.5 times more likely to die than single-born lambs, predominantly due to differences in lamb birth weight (BW) [8]. Twin lambs are born lighter [9] and typically exhibit reduced thermoregulatory capacity due to poor levels of energy stores at birth [10], delaying the attainment of behavioural milestones critical for neonatal survival. Consequently, the risk of mortality due to starvation and cold exposure is increased [11,12]. A summation of studies suggests that the most practical and economic way of improving twin lamb survival to weaning is through maternal nutritional supplementation during pregnancy [8,13,14].

Betaine is an amino acid derivative that acts as a methyl donor and has the capacity to increase the production of creatine in the brain and muscle tissue of animals, with creatine identified as an endogenous neuroprotectant [15,16,17]. These beneficial effects of creatine suggest that betaine has the potential to improve twin lamb survival by increasing energy stores in the lamb at birth and protecting the brain from hypoxic birth injury, leading to improvements in lamb BW, thermoregulation, and vigour. Maternal betaine supplementation during pregnancy in sows increased the number of piglets born alive [18] and piglet energy synthesis and glycogen stores at birth [19]. Further, our previous study demonstrated that supplementing 4 g/day of dietary betaine to twin-bearing Merino ewes during the second half of pregnancy improved twin lamb survival to seven days of age and shortened the interval from birth to first successful suck from the udder [20]. Whilst this experiment was conducted in an indoor facility and provided favourable conditions to maximise lamb survival, significantly fewer lambs in the 4 g betaine treatment died in the first three days post-partum (pp) compared to the two other treatment groups. Therefore, supplementing 4 g/day of dietary betaine may produce more profound effects when ewes lamb in more challenging environmental conditions. Thus, we examined the effects of supplementing twin-bearing ewes with 4 g/day of dietary betaine during late gestation until ~49 days pp on maternal and neonatal lamb creatine and creatinine concentrations and lamb viability measures and survival in extensive grazing systems.

## 2. Materials and Methods

This project was approved by the Primary Industries and Regions South Australia Animal Ethics Committee (approval number: #01/22) and conducted in accordance with the Australian Code of Practice for the Use of Animals for Scientific Purposes (8th Edition; 2013).

### 2.1. Location and Animal Management

This experiment was conducted during the autumn/winter of 2022 at the South Australian Research and Development Institute’s Kybybolite Research Centre, Kybybolite, South Australia (36.87° S, 140.93° E). A total of 1419 mature (3–7 years of age), multiparous South Australian purebred Merino ewes (*Ovis aries*) were treated with 14 mL of Avomec Duel Sheep Drench (Boehringer Ingelheim Animal Health Australia Pty Ltd., North Ryde, NSW, Australia) prior to being housed in paddocks with Border Leicester rams for three oestrous cycles (~50 day mating period) commencing early January. Ewes were pregnancy scanned via transabdominal ultrasonography by a commercial technician at 70 days after ram introduction using an Ovi-Scan 6 Ultrasound (axial sector sheep probe, 3.5 MHz 170° view; BCF^™^ Ultrasound, Mitcham, VIC, Australia) to determine pregnancy status, litter size, and foetal age. Foetal age was determined by measuring the diameter of the body against measurement bars on the Ovi-Scan. Foetal ageing was used to classify ewes that were conceived at the first, second, or third oestrous during the mating period and were therefore defined as ‘early mated’, ‘mid mated’ or ‘late mated’ pregnancies, respectively. Furthermore, the approximate day of gestation was determined by averaging the estimated mating dates of the early, mid, and late mated pregnancies. Ewes were managed on pasture and received barley grain (~400 g/ewe) three times per week and a bale of oat hay weekly (~4.2 kg/ewe/week) to meet nutritional and energy requirements [21]. The quantity of barley grain given to the ewes was adjusted throughout the experiment based on measures of average ewe live weight (LW), body condition score (BCS), and stage of gestation. Ewe LW and BCS [22] were measured on dG 70, 110, 130, and at ~49 and ~93 days pp. All paddocks used in this study were of similar size (~9.5 ha), topography, shelter, and feed availability and quality. Each paddock contained a mixture of subterranean clover (*Trifolium subterraneum*), annual ryegrass (*Festuca perennis*), barley grass (*Hordeum vulgare*), and phalaris (*Phalaris aquatica*). Ewes had *ad libitum* access to clean drinking water. Daily meteorological data were obtained from the Kybybolite Research Centre weather station during the ~28-day lambing observation period (1st–29th June). These included ambient temperature (°C), relative air humidity (%), wind speed (km/h), rainfall (mm), and chill index factor (kJ m^−2^ h^−1^), which are detailed in Table 1. The chill index factor was used as a model for estimating heat loss from lambs during the first day of life and was based on the cooling effects of temperature, wind speed, and rain [23,24] and is as follows:

*C* = (11.7 + 3.1 *v*^0.5^) (40 − *T*) + 481 + *R* (1)

where *C* is the potential heat loss (kJ m^−2^ h^−1^), *v* is the mean daily wind velocity (m s-1), *T* is the mean daily temperature (°C), and *R* = 418 (1 − e^−0.04x^), where *x* is the daily rainfall (mm).

### 2.2. Animals and Nutritional Treatments

A total of 230 twin-bearing ewes were randomly assigned to one of two treatment groups based on foetal age, LW and BCS: (1) control: 0 g of betaine/ewe/day throughout gestation (CTL); or (2) betaine: 4 g of betaine/ewe/day from dG 110 until ~49 days pp (BET), with two replicates for each treatment group. Ewes that were conceived in the first and second oestrous only were selected in order to refine the length of the supplementation and lambing period. The number of pregnant ewes per treatment group is detailed in Table 2. Following selection, ewes were separated into their respective treatment groups, moved to their experimental paddocks, and conditioned to consume their supplemental pelleted feed ration through commercial lick feeders (Bromar Engineering, Grenfell, NSW, Australia) in preparation for the commencement of experimental diets, which began on dG 110. The CTL ewes received a common basal pelleted diet (Laucke Mills, Daveyston, SA, Australia), whilst the BET ewes received a betaine-supplemented pellet at a dose rate of 32.5 L/tonne (Liquid Natural Betaine 38% intermediate bulk container, Feedworks, Lancefield, VIC, Australia). The selected dose rate and administration time were based on our previous work in twin-bearing Merino ewes [20]. Whilst the ewes had continuous access to the supplemental pellets contained in the lick feeders for the duration of the experiment, the flow rate of the feeders was set to deliver approximately 400 g/ewe/day to achieve the target dose of 4 g of betaine/ewe/day. On dG 130, ewes were fitted with unique collared numbers to allow for easy identification during the lambing observation period. Feed on offer in the field (FOO; kg dry matter (DM)/ha) was determined by taking ten calibration cuts from each individual paddock using a 0.1 m^2^ quadrat (i.e., 10 cuts/paddock) on dG 80, 110, 130, and at ~49 days pp. Pasture biomass from each quadrat was harvested with a set of shears to ground level, and soil and foreign matter were removed. Pasture samples were then weighed, oven dried to a constant weight at 70 °C, and then re-weighed to determine DM content.

### 2.3. Measures of Lamb Viability Collected within 4–24 h after Birth

Starting on day 147 (dG 1 = ram entry), ewes were monitored between 0800–1200 and 1300–1730 h each day. Newborn lambs were ear-tagged when estimated to be at least 4 h old to minimise the potential of mismothering or, if born overnight, were processed the next morning. Lamb measurements collected within 4–24 h pp included date of birth, sex, BW, rectal temperature, meconium staining score (MSS) [20] and vigour score (VS; adapted from [25]). The vigour score was assessed when this researcher pursued the lambs for data collection. Upon release from this researcher, three timed lamb behaviours were determined by a single researcher and included time to contact the ewe, time to first suckle, and time to follow the ewe (adapted from [25]). Approximate gestation length, difficulty of parturition [20], and maternal behaviour score (MBS; [26]; and adapted from [27]) were also collected within 4–24 h pp. Ewes were allowed to deliver and mother their lambs without intervention unless obstetric assistance was required. Details of the measurements and scoring systems for traits recorded for newborn lambs and ewes are given in Table 3. Lambs were weighed, de-tailed, castrated, and vaccinated with 1.0 mL of GlanEry^®^ 7 in 1 B12 Vaccine for Sheep, 0.02 mL of Scabigard^®^ Vaccine, and 1.0 mL of Gudair^®^ Vaccine (females only) (vaccines listed were from Zoetis Australia Pty Ltd., Rhodes, NSW, Australia) at 49.1 ± 7.2 days of age. At 93.1 ± 7.2 days of age, lambs were weighed and weaned. Average daily gain from birth to 49 days pp and birth to 93 days pp was determined by dividing the weight of each individual by their age at each time point and is expressed in g/day. Lamb survival was calculated based on the number of lambs that were alive at birth and at 3, 49, and 93 days pp. Also, lamb survival occurring during the first three days pp was related to chill index factor, with details of index determination given above. The effect of treatment on lamb survival was examined when chill index factor was either low (<1000 kJ m^−2^ h^−1^) or high (>1000 kJ m^−2^ h^−1^), as justified by [28]. Lamb necropsies, where possible, were performed to determine cause of death (Table 3) of any lambs found dead or were euthanized using the methods described by [6,29]. Lambs that were absent at 49 days pp. or 93 days pp. for which no carcass were found were classified as ‘unknown’.

### 2.4. Ewe and Lamb Blood Sampling and Processing

A 10 mL blood sample was collected via jugular venepuncture directly into a lithium heparin tube (BD Vacutainer^®^, Oakville, ON, Canada) using an 18 G vacutainer needle from a subset of ewes on dG 110 (CTL, *n* = 39; BET, *n* = 36). These ewes were randomly selected from each treatment group and stratified according to LW and BCS. Samples were collected to establish baseline creatine and creatinine profiles prior to the commencement of feeding experimental diets. Another 10 mL blood sample was collected from the same subset of ewes on dG 130 using the same procedure as above. Within 4–24 h pp, 5 mL of blood was collected from each lamb using jugular venepuncture procedures and a 21 G needle and 5 mL syringe. Glucose concentration (5–10 µL) was measured immediately (Abbott Freestyle Optium Neo device, Melbourne, VIC, Australia), which was validated and calibrated as described by [20]. Remaining blood was equally distributed into a lithium heparin blood tube and a clot activator blood tube. Ewe and lamb blood samples collected into lithium heparin tubes were centrifuged at 1509× *g* for 15 min within 2 h of collection, after which plasma was evenly distributed into two 1.5 mL tubes and stored at −20 °C for later analyses of creatine and creatinine concentrations. The lamb clot activator tubes were stored at 4 °C for 24 h, centrifuged, and serum was stored at −20 °C for later analysis of immunoglobulin G (IgG) concentration. Lamb serum IgG concentrations were determined by a previously validated radial immunodiffusion assay [20].

### 2.5. Creatine and Creatinine Concentration Analyses

Circulating creatine and creatinine concentrations were determined in ewe plasma samples collected on dG 110 and dG 130 (at both collection points: CTL, *n* = 39; BET, *n* = 36) and lamb plasma samples collected within 4–24 h pp (CTL, *n* = 74; BET, *n* = 61) at Medizinisches Labour Bremen GmbH (Bremen, Germany). Briefly, plasma samples were diluted with water, and an acetonitrile/methanol mixture (500 mL) was then added to each sample along with the addition of 50 µL of an internal standard (10 and 25 mmol/L for [^2^H_3_]-creatine and [^2^H_3_]-creatinine, respectively). Samples were then mixed and centrifuged at 16,278× *g* for 3 min. Following centrifugation, aliquots of supernatant (200 µL) were transferred to a 96-well microtitre plate for analysis. Creatine (mg/dL) was immediately determined by high-performance liquid chromatography-mass spectrometry using known aqueous standards. Creatinine (mg/dL) was determined by an enzymatic test using the Cobas^®^ 8000 modular analyser series machine (Roche Diagnostics International Ltd., Rotkreuz, Switzerland).

### 2.6. Statistical Analysis

Statistical analyses were conducted in IBM SPSS Statistics 29 software (Version 29.0, IBM Corp., Armonk, NY, USA). A Pearson’s chi-square test was used to analyse the effects of betaine treatment on lamb survival at each time point, chill index factor on lamb survival at three days pp, and all scoring systems. Causes of lamb mortality were analysed by Fisher’s exact test. Effects of treatment on all single time point measures collected from the ewe and lamb (non-repeat) were analysed by a general linear model, with fixed factors of betaine treatment and two-way interactions of treatment with replicate and sex (lamb data only). Outcomes measured multiple times (ewe LW and BCS, FOO and lamb LW) were analysed using a generalised linear mixed model, with fixed factors of treatment, time of collection, replicate, and sex (lamb data only), with individual ewe ID fitted in the model as a random effect. Description of the data indicated that data distribution was normal (Shapiro–⁠Wilks’s test) and variances were homogenous (Levene’s test). Data were checked for outliers. Data from ewes that were not pregnant (*n* = 6), delivered singles (*n* = 13) or triplets (*n* = 1), and their corresponding lambs (*n* = 16) were excluded from the analysis. Final analyses therefore included *n* = 110 CTL ewes and *n* = 103 BET ewes. Significance was accepted when *p* ≤ 0.05, with *p* < 0.1 considered a trend.

## 3. Results

### 3.1. Lamb Survival, Chill Index Factor, and Cause of Death

Maternal betaine supplementation had no effect on twin lamb survival at birth, 3 days pp, or at 49 days pp and 93 days pp (Table 4). There was no effect of treatment on lamb survival at three days pp when chill index factor was either low (<1000 kJ m^−2^ h^−1^) or high (>1000 kJ m^−2^ h^−1^) (Table 4). Lamb survival was lower when chill index factor was high (>1000 kJ m^−2^ h^−1^) (77.2% vs. 89.7%; *p* < 0.001). The proportion of lambs that died due to primary predation was greater in the BET treatment than the CTL treatment (*p* < 0.05; Table 4). The major cause of lamb deaths across both treatment groups was attributed to parturition-related issues.

### 3.2. Neonatal Lamb Viability Measures

Supplementation of betaine had no significant effect on lamb LW at birth (Table 5); however, CTL lambs were heavier than BET lambs at 49 days pp (*p* < 0.05; Table 5), but this difference was not observed at 93 days pp. There was a significant treatment effect on lamb ADG from birth to 49 days pp, whereby CTL lambs grew faster than BET lambs (*p* < 0.05; Table 5); however, this effect was not observed for ADG from birth to 93 days pp. None of the interactions of betaine treatment with other fixed effects were significant for lamb weight and ADG between all time points. BET lambs had a higher rectal temperature within 4–24 h following birth than CTL lambs (*p* < 0.05). The time taken after release from this researcher to first suckling was quicker in the CTL lambs than BET lambs (*p* < 0.05; Table 5). Vigour score, MSS, time to contact the ewe, and time to follow the ewe were unaffected by treatment and first-order interactions.

### 3.3. Ewe and Lamb Blood Chemistry Analysis

CTL ewes tended to have higher creatine concentrations than BET ewes on dG 110 (*p* < 0.1); however, on dG 130, BET ewes had higher creatine concentrations compared to CTL ewes (*p* < 0.05; Table 6). On dG 110, there was a tendency (*p* < 0.1) for creatinine concentration to be higher in the BET ewes compared to the CTL ewes (Table 6), with creatinine concentrations being higher in the BET ewes than CTL ewes on dG 130 (*p* < 0.05; Table 6). From dG 110 to 130, the CTL ewes experienced a decrease in creatine concentration, whereas the BET ewes experienced an increase in creatine concentrations (*p* < 0.05; Table 6). CTL ewes experienced a greater decrease in creatinine concentrations from dG 110 to 130 compared with the BET ewes (*p* < 0.05; Table 6). Further, a treatment × replicate interaction (*p* = 0.008) was also observed, whereby the BET replicate one ewes had a lower decrease in creatinine concentrations from dG 110 to 130 than CTL replicate one and two ewes but not BET replicate two ewes (Estimated means ± SEM for replicate one CTL and BET ewes were −0.20 ± 0.03 and −0.01 ± 0.02 mg/dL, respectively; Estimated means ± SEM for replicate two CTL and BET ewes were −0.29 ± 0.03 and −0.24 ± 0.03 mg/dL, respectively). Similarly, there was a significant treatment × replicate interaction (*p* = 0.002) for lamb glucose concentration, where BET replicate two lambs had a higher plasma glucose than BET replicate one and CTL replicate two lambs (Estimated means ± SEM for replicate one CTL and BET lambs were 5.66 ± 0.22 and 5.26 ± 0.23 mmol/L, respectively; Estimated means ± SEM for replicate two CTL and BET lambs were 5.06 ± 0.23 and 6.02 ± 0.24 mmol/L, respectively). Maternal betaine supplementation had no effect on lamb creatine, creatinine, and serum IgG concentrations within 4–24 h pp (Table 6).

### 3.4. Ewe Live Weight, Body Condition Score, Feed on Offer, Approximate Gestation Length, Difficulty of Parturition, and Maternal Behaviour Score

Dietary treatments had no effect on ewe LW at any time point (Table 7). BET ewes had a higher BCS than CTL ewes at 93 days pp (*p* < 0.05; Table 7), but this did not differ between treatments at any other time points (Table 7). Feed on offer tended to be higher in BET paddocks compared to CTL paddocks at dG 110 (*p* < 0.1; Table 7). However, CTL paddocks tended to have higher FOO recorded at 49 days pp than BET paddocks (*p* < 0.1; Table 7). There were no significant treatment × replicate interactions for FOO at all time points. CTL ewes had a longer gestation length compared to BET ewes (*p* < 0.05; Table 7). Furthermore, there was also a significant interaction (*p* < 0.001) between treatment and replicate for gestation length, where CTL replicate one ewes had a longer gestation length than BET replicate one and CTL and BET replicate two ewes (Estimated means ± SEM for replicate one CTL and BET ewes were 151.5 ± 0.6 and 147.4 ± 0.6 days, respectively; Estimated means ± SEM for replicate two CTL and BET ewes were 147.2 ± 0.6 and 147.9 ± 0.6 days, respectively). There tended to be a higher proportion of CTL ewes that received a MBS score of 4 and 5 than the BET ewes (*p* < 0.1; Table 7). Difficulty of parturition was unaffected by betaine treatment.

## 4. Discussion

To the best of our knowledge, this study is the first to determine whether maternal supplementation with 4 g/day of dietary betaine during late gestation until 49 days pp would improve twin lamb weight, thermoregulatory capacity, vigour, and survival from birth to weaning in flocks lambing under extensive grazing conditions.

One major finding from this study was that we observed differences in maternal creatine concentration following betaine supplementation. More specifically, ewes supplemented with 4 g/day of betaine for 20 days had ~16% higher plasma creatine concentrations on dG 130 and experienced a greater change in creatine concentrations from dG 110 to 130 compared with CTL ewes. In the transmethylation pathway, betaine donates a labile methyl group, which facilitates the reformation of methionine from homocysteine. Methionine is then converted to *S*-adenosyl methionine, a compound that, during degradation, provides the final methyl group for guanidinoacetic acid for the synthesis of creatine [15,16,30,31]. It is, therefore, suggested that supplementing dietary betaine provided more methyl groups, which in turn effectively enhanced methionine availability for creatine production. As such, the observed increase in creatine in the BET ewes is not surprising. In addition to creatine, we observed differences in plasma creatinine concentrations between treatment groups, whereby the BET ewes had ~12% higher concentrations recorded on dG 130 compared to the CTL ewes. Creatinine is universally known as the breakdown product of creatine phosphate—the phosphorylated form of creatine—in muscle tissue and is typically produced at a constant rate by the body [32]. Due to a betaine-induced increase in creatine concentrations, it was hypothesised that supplementing betaine would also lead to subsequent increases in plasma creatinine concentrations as a result of greater creatine phosphate catabolism. For example, studies conducted in canines and rats demonstrated that supplementing creatine increased circulating creatine concentrations, which consequently led to increased circulating creatinine [33,34]. Since the BET ewes had higher creatine concentrations recorded on dG 130, it is not surprising that we observed subsequent increases in plasma creatinine concentrations as well. However, one limitation of the current experiment was that due to calibration issues with the weigh scales for the lick feeders, the consumption rate of the experimental diets for each treatment group was based on an estimation and thus needs to be considered in future methodology. Despite this, to the best of our knowledge, this is the first study to demonstrate that feeding betaine during late gestation can increase plasma creatine and creatinine concentrations in twin-bearing Merino ewes under extensive grazing systems.

Interestingly, twin lambs born to ewes supplemented with 4 g/day of dietary betaine were 0.4% warmer than the CTL lambs within the first 4–24 h following birth. Non-shivering thermogenesis is a vital physiological mechanism that generates instantaneous heat to prevent hypothermia and, as such, significantly influences the survival of the lamb immediately after birth [35]. The principal source of non-shivering thermogenesis in neonatal lambs is brown adipose tissue, which constitutes 2.0–4.5% of lamb body weight [12,36,37]. As a methyl donor, betaine has the potential to improve thermoregulation in neonatal lambs by increasing circulating creatine concentrations [16]. Creatine plays a critical role in adipose tissue thermogenesis with its primary action in regulating adenosine diphosphate and adenosine triphosphate metabolism in mitochondrial cells [38,39,40]. Since the intracellular creatine pool used in thermogenesis is regulated by influxes from the circulatory system of an animal [38], we hypothesised that heat production in the BET lambs would be greater at birth, potentially due to a betaine-induced increase in creatine. However, this was not supported by the current data for lamb creatine concentrations collected within 4–24 h after birth, as there were no treatment differences. Since this study was conducted under field conditions to reflect commercial management systems of sheep in Australia, we cannot dismiss the possibility of external factors potentially influencing this result, including the delay between birth and blood collection. Because the ewes lambed outdoors, we were unable to collect blood samples from the lambs close to birth, and thus, the current data may have been affected by suckling behaviour as some colostrum components (i.e., proteins, vitamins, minerals, growth factors, etc.) can influence circulating metabolites (i.e., creatine) in neonatal lambs following ingestion [41,42]. It is, therefore, possible that the variation in time of collection and the potential impact of suckling may have masked any treatment effects on circulating creatine concentrations in the lambs; however, this is only a suggestion and not known. Despite this, the improvement in twin lamb rectal temperature under field conditions, where lamb heat loss/chill index factor did affect survival, is promising.

It was anticipated that maternal betaine supplementation would improve lamb BW due to the action of betaine to improve growth and feed efficiency of an animal via increased intestinal cell surface area for nutrient digestibility and utilisation [16,43]. However, as mentioned earlier, neither BW nor subsequent weight at 49 or 93 days pp were improved in the BET lambs. Interestingly, whilst there were no improvements in BW of lambs born to betaine-supplemented ewes, the BET ewes had a shorter gestation length compared to the CTL lambs. A typical gestational period of a twin-bearing Merino ewe ranges between approximately 147 and 151 days [20,44,45,46]. Despite both treatment groups falling within this range, the BET ewes had approximately two days less than the CTL ewes for foetal growth and development in utero, yet the BET lambs were as heavy as the CTL lambs at birth. Previous research has shown that betaine has the potential to influence nutrient transport and epigenetic modifications of foetal genes [47,48]. In this study by Jin et al. [47], piglets born from sows fed 3 g of betaine/kg daily from mating until parturition had greater BW when compared to the control piglets. The authors attributed this improvement to epigenetic modifications that resulted in an increased expression of serum insulin-like growth factor-1 content—a well-known gene involved in the regulation of foetal development [49]. While not directly analysed in the current study, it is possible that maternal betaine supplementation may have enhanced this epigenetic effect in the BET lambs in utero and thus could explain why these lambs had a similar BW to the CTL lambs despite a shorter gestational period. More research is required to further elucidate this physiological mechanism. It is important to note, however, that gestation length was derived from an estimate of foetal age (the exact mating dates for each individual were not recorded), and though seen as a limitation of the data, the estimated difference was highly significant (*p* = 0.004).

There were differences detected in LW at 49 days pp, whereby the CTL lambs were ~5% heavier than the BET lambs, an unexpected finding that could be related to the effects of a high nutritional plan on ewe lactation performance. Nutrition of the ewe during lactation significantly influences lamb survival and growth to weaning [8,50,51]. More specifically, sub-maintenance nutrition during this period has been strongly linked to decreases in daily milk yield and length of the lactation period [52,53,54], thereby resulting in subsequent decreases on lamb LW and LW gain [55,56]. In addition, Corner-Thomas et al. [57] reported that lambs born to ewes that had access to pasture with high FOO values (i.e., 1500 to 1700 kg DM/ha) during lactation were heavier at weaning compared to lambs born from ewes exposed to pasture with lower FOO values, suggestive of greater milk production in the ewes. While pasture FOO in the current experiment was much lower (~390 to 830 kg DM/ha) than in this study of Corner-Thomas et al. [57], our ewes also received the experimental pellet diet in addition to barley grain and hay, thereby meeting the nutritional demands of these animals. Despite the BET ewes having access to the experimental pellets, it is speculated that the combination of higher pasture FOO in the CTL paddocks and experimental pellets may have resulted in greater milk production during the early lactation period in these animals. Consequently, this may have contributed to the CTL lambs growing faster from birth to 49 days pp and therefore being heavier at 49 days pp than the BET lambs. Furthermore, the CTL lambs were quicker to suckle from the udder after release from this researcher than the BET lambs, highlighting that these lambs may have had greater suckling periods and resulted in increased milk ingestion and growth rates. In contrast, lack of a treatment difference in ewe LW and BCS at 49 days pp would suggest that nutrient intake of the ewes during early lactation was similar between treatments and that the lower LW of BET lambs is inexplicable.

## 5. Conclusions

Overall, this study indicated that supplementing the diets of twin-bearing Merino ewes with 4 g/day of betaine from dG 110 increased maternal creatine and creatinine concentrations at dG 130 and rectal temperature of lambs within 4–24 h pp, indicating greater thermoregulatory ability. While these findings demonstrate some promising preliminary outcomes in the field, this nutritional approach did not improve twin lamb survival. As such, further research is required before maternal betaine supplementation during late gestation can be used as an on-farm strategy to improve lamb viability and survival.

## Figures and Tables

**Table 1:** Weather conditions during the lambing observation period. Data are estimated marginal means ± standard error of the mean (SEM).

Meteorological Measure	Mean	Range
Ambient temperature, °C	10.2 ± 0.3	7.6–14.0
Relative air humidity, %	86.7 ± 1.0	77.0–96.2
Wind speed, km/h	7.3 ± 0.6	1.8–15.3
Rainfall, mm/day	1.8 ± 0.7	0–17.6
Chill index factor, kJ m^−2^ h^−1^	983.4 ± 12.1	881.4–1225.0

**Table 2:** Number of twin-bearing Merino ewes receiving either CTL or BET ^^^ supplemented diets per replicate based on early and mid-mated pregnancies.

Treatment Group		Number of Ewes		
		Early-Mated	Mid-Mated	Total
CTL	Replicate 1	58	0	58
	Replicate 2	32	25	57
BET	Replicate 1	58	0	58
	Replicate 2	29	28	57

^^^ CTL: Control; BET: Betaine.

**Table 3:** Measurement and scoring systems for newborn lamb and ewe traits and death categories of perinatal lambs used in the current study.

Measure/Score	Description
Difficulty of parturition	Score range 1–3, where 1 is no assistance required, 2 is minor assistance given, and 3 is lamb delivered manually [20].
Meconium staining score, MSS	Score range 1–4, where 1 is no staining, 2 is light, 3 is moderate, and 4 is severe [20].
Vigour score, VS	Score range 1–5, where 1 is lamb in good condition, energetic, runs very easily when pursued, takes >30 s to catch, and 5 is lamb weak, laying on ground, unable to hold head up, slow breathing, cold body temperature [25].
Time-based lamb behaviours	Based on time taken from release to contact the ewe, first suckle upon return and follow the ewe [25].
Maternal behaviour score, MBS	Subjective measure of ewe mothering ability; score range 1–5, where 1 (best) is ewe stays close to the lambs during tagging and 5 (worst) is ewe flees, shows no interest in the lambs, and does not return [26,27].
**Death category [6,29]**	
Parturition-related	Dystocia: oedema, cranial and CNS haemorrhage; not walked; not breathed. Stillborn: significant cranial and CNS haemorrhage, fat not metabolised ± breathed. Birth injury: significant cranial and CNS haemorrhage, breathed, fat metabolised, ± empty stomach, <5 days old. Note: The degree of meconium staining on the fleece of lambs and its presence in the upper airway were not examined.
Birth defect	Signs of abnormalities, i.e., deformed head, born with no anus.
Starvation/mismothering	Null to cranial and CNS haemorrhage, fat metabolised, breathed, cleaned, walked, empty stomach.
Primary cold exposure	Yellow subcutaneous oedema; fat metabolised; breathed; walked.
Primary predation	No signs of abnormality or injury at birth; significant bleeding from fatal predatory wounds.
Infection	Lesions on the lungs or liver sucked; no additional samples were taken to identify causative organisms.
Unknown	Lamb missing.

**Table 4:** Cumulative survival from birth to 93 days post-partum (pp), effect of chill index factor (CIF) on survival, and causes of death of twin lambs born from Merino ewes receiving either CTL or BET ^ supplemented diets. Data are a percentage, and numbers in parentheses indicate the number of alive lambs or lambs that died within each death category for each individual treatment, respectively.

Survival (%) at:	Treatment		*X* ^2^	*p*-Value
	CTL	BET		
*n*	220	206		
Birth	95.5 (210)	95.6 (197)	0.008	0.930
3 days pp	83.6 (184)	88.3 (182)	1.953	0.162
~49 days pp	78.2 (172)	80.6 (166)	0.374	0.541
~93 days pp	75.0 (165)	79.1 (163)	1.023	0.312
**CIF and survival at 3 days pp**				
*n*	218	206		
Low (<1000 kJ m^−2^ h^−1^)	87.5 (152)	91.8 (158)	1.527	0.217
High (>1000 kJ m^−2^ h^−1^)	77.3 (66)	77.1 (48)	0.001	0.981
**Causes of death (%) ^+^**				
*n*	55	43		
Parturition-related	41.8 (23)	32.6 (14)	0.881	0.348
Birth defect	5.5 (3)	4.7 (2)	0.032	0.858
Starvation/mismothering	12.7 (7)	14.0 (6)	0.032	0.859
Primary cold exposure	1.8 (1)	0.0 (0)	0.790	0.374
Primary predation	0.0 (0) ^a^	14.0 (6) ^b^	8.175	0.004
Infection	1.8 (1)	4.7 (2)	0.653	0.419
Unknown	36.4 (20)	30.2 (13)	0.902	0.278

^^^ CTL: Control; BET: Betaine. ^+^ Proportions consist of deceased lambs only. ^ab^ Superscripts indicate significant differences (*p* < 0.05) within a row.

**Table 5:** Mean live weight (LW), average daily gain (ADG), meconium staining score (MSS), vigour score (VS), rectal temperature, and timed behavioural responses of twin lambs born from Merino ewes receiving either CTL or BET ^^^ supplemented diets. Data are estimated marginal means ± SEM and a percentage with numbers in parentheses.

Measure	Treatment		*p*-Value
	CTL (*n*)	BET (*n*)	
LW, kg			
Birth	5.10 ± 0.25 (197)	5.08 ± 0.26 (180)	0.957
~49 days pp	17.24 ± 0.27 (170) ^a^	16.45 ± 0.27 (164) ^b^	0.036
~93 days pp	29.20 ± 0.27 (161)	29.11 ± 0.27 (162)	0.819
ADG, g/day			
Birth—49 days pp	254.6 ± 5.0 (170) ^a^	231.9 ± 5.1 (164) ^b^	0.002
Birth—93 days pp	274.3 ± 6.3 (161)	259.7 ± 6.3 (162)	0.102
MSS, 1–4 ^+^, % (*n*) 1	38.1 (80)	36.3 (70)	0.903
2	34.3 (72)	36.3 (70)	
3	27.6 (58)	27.4 (53)	
4	0 (0)	0 (0)	
VS, 1–5 ^#^, % (*n*) 1	36.1 (75)	31.6 (60)	0.387
2	27.9 (58)	37.4 (71)	
3	30.3 (63)	26.3 (50)	
4	5.3 (11)	4.2 (8)	
5	0.4 (1)	0.5 (1)	
Rectal temperature, °C	39.10 ± 0.05 (205) ^a^	39.25 ± 0.05 (185) ^b^	0.023
Time to contact the ewe, s	34.98 ± 5.57 (161)	38.12 ± 5.68 (152)	0.693
Time to first suckle, s	23.19 ± 7.51 (39) ^a^	49.42 ± 7.39 (38) ^b^	0.016
Time to follow the ewe, s	21.15 ± 2.51 (100)	19.79 ± 2.36 (111)	0.694

^^^ CTL: Control; BET: Betaine. ^ab^ Superscripts indicate significant differences (*p* < 0.05) within a row. ^+^ 1: no staining; 2: light; 3: moderate; 4: severe. ^#^ 1: lamb in good condition, energetic, runs very easily when pursued, takes >30 s to catch; ^#^ 5: lamb is weak, laying on ground, unable to hold head up, slow breathing, cold body temperature.

**Table 6:** Mean plasma creatine and creatinine concentrations of ewes at days 110 and 130 of gestation receiving either CTL or BET ^^^ supplemented diets and plasma creatine, creatinine, glucose, and serum immunoglobulin G (IgG) concentrations of lambs within 4–24 h post-partum. Data are estimated marginal means ± SEM with numbers in parentheses.

Measure	Treatment		*p*-Value
	CTL (*n*)	BET (*n*)	
*Ewes*			
Creatine, mg/dL			
dG 110	2.87 ± 0.07 (39) ^x^	2.69 ± 0.08 (36) ^y^	0.096
dG 130	2.42 ± 0.10 (36) ^a^	2.84 ± 0.10 (36) ^b^	0.003
Change from dG 110 to 130	−0.46 ± 0.10 (36) ^a^	0.16 ± 0.10 (36) ^b^	<0.001
Creatinine, mg/dL			
dG 110	0.87 ± 0.02 (39) ^x^	0.91 ± 0.02 (36) ^y^	0.073
dG 130	0.66 ± 0.02 (36) ^a^	0.74 ± 0.02 (36) ^b^	0.009
Change from dG 110 to 130	−0.25 ± 0.02 (36) ^a^	−0.12 ± 0.02 (36) ^b^	<0.001
*Lambs*			
Creatine, mg/dL	2.34 ± 0.14 (74)	2.52 ± 0.15 (61)	0.371
Creatinine, mg/dL	1.03 ± 0.05 (74)	1.02 ± 0.05 (61)	0.830
Glucose, mmol/L	5.40 ± 0.16 (194)	5.65 ± 0.17 (179)	0.284
Serum IgG, mg/mL	29.91 ± 1.34 (171)	31.01 ± 1.39 (158)	0.567

^^^ CTL: Control; BET: Betaine. ^ab^ Superscripts indicate significant differences (*p* < 0.05) within a row. ^xy^ Superscripts indicate trend (*p* < 0.1) within a row.

**Table 7:** Mean live weight (LW), body condition score (BCS), feed on offer (FOO), approximate gestation length, difficulty of parturition, and maternal behaviour score (MBS) of Merino ewes receiving either CTL or BET ^^^ supplemented diets. Data are estimated marginal means ± SEM and a percentage value with numbers in parentheses.

Measure	Treatment		*p*-Value
	CTL (*n*)	BET (*n*)	
*Ewe LW*, kg			
dG 70	64.64 ± 0.71 (110)	65.01 ± 0.73 (102)	0.715
dG 110	66.69 ± 0.71 (110)	66.61 ± 0.73 (102)	0.955
dG 130	70.26 ± 0.71 (110)	69.37 ± 0.73 (103)	0.383
~49 days pp	61.68 ± 0.72 (101)	62.21 ± 0.74 (97)	0.611
~93 days pp	68.27 ± 0.72 (101)	68.55 ± 0.74 (99)	0.792
*Ewe BCS*, 0–5			
dG 70	2.92 ± 0.10 (108)	2.97 ± 0.10 (101)	0.682
dG 110	3.04 ± 0.10 (110)	3.05 ± 0.10 (102)	0.939
dG 130	2.92 ± 0.10 (110)	2.84 ± 0.10 (103)	0.582
~49 days pp	2.42 ± 0.10 (101)	2.48 ± 0.10 (97)	0.672
~93 days pp	3.01 ± 0.10 (101) ^a^	3.35 ± 0.10 (99) ^b^	0.018
*FOO*, kg DM/ha			
dG 90	515.3 ± 77.5 (19)	507.5 ± 75.5 (20)	0.943
dG 110	389.5 ± 75.5 (20) ^x^	587.0 ± 75.5 (20) ^y^	0.066
dG 130	536.0 ± 75.5 (20)	397.0 ± 75.5 (20)	0.195
~49 days pp	831.0 ± 75.5 (20) ^x^	663.2 ± 75.5 (20) ^y^	0.097
Approximate gestation length, days	149.3 ± 0.4 (109) ^a^	147.6 ± 0.4 (103) ^b^	0.004
Difficulty of parturition, 1–3 ^+^, % (*n*) 1	100.0 (210)	97.9 (189)	0.111
2	0.0 (0)	0.5 (1)	
3	0.0 (0)	1.6 (3)	
MBS, 1–5 ^#^, % (*n*) 1	13.2 (26)	10.3 (20)	0.059
2	27.2 (53)	24.7 (48)	
3	22.6 (44)	36.1 (70)	
4	26.2 (51)	19.6 (38)	
5	10.8 (21)	9.3 (18)	

^^^ CTL: Control; BET: Betaine. ^ab^ Superscripts indicate significant differences (*p* < 0.05) within a row. ^xy^ Superscripts indicate trend (*p* < 0.1) within a row. ^+^ 1: no assistance; 2: minor assistance (correct lamb posture); 3: major assistance (manual pulled lamb). ^#^ 1: Ewe stays close to the lambs during tagging (best); ^#^ 5: Ewe flees, shows no interest in the lambs, and does not return (worst).

## Data Availability

The original contributions presented in this study are included in the article; further enquiries can be directed to the corresponding author.
